# Occlusion Avoidance for Harvesting Robots: A Lightweight Active Perception Model

**DOI:** 10.3390/s26010291

**Published:** 2026-01-02

**Authors:** Tao Zhang, Jiaxi Huang, Jinxing Niu, Zhengyi Liu, Le Zhang, Huan Song

**Affiliations:** School of Mechanical Engineering, North China University of Water Resources and Electric Power, Zhengzhou 450011, China; zhangtao@ncwu.edu.cn (T.Z.); 18607959553@163.com (J.H.); lzy2095254304@gmail.com (Z.L.); zl000803@163.com (L.Z.); songhuan@ncwu.edu.cn (H.S.)

**Keywords:** harvesting robot, occlusion avoidance, lightweight YOLOv8n, active per ception strategy

## Abstract

**Highlights:**

**What are the main findings?**

- A lightweight YOLOv8n model integrated with C2f-FasterBlock and SE attention achieves high apple detection accuracy (mAP = 0.885) and real-time performance (83 FPS) with 37% fewer parameters and a compact 4.3 MB size
- An end-to-end active perception framework based on ResNet50 and multi-modal fusion enables the robotic arm to autonomously navigate to optimal viewpoints, significantly reducing occlusion and improving recognition success.

**What are the implications of the main findings?**

- The proposed co-design of efficient perception and active sensing offers a practical solution for reliable fruit detection in cluttered orchard environments, addressing a key bottleneck in agricultural automation.
- The system’s direct mapping from visual input to motion planning demonstrates a scalable paradigm for closed-loop robotic harvesting, paving the way for deployment in real-world field conditions.

**Abstract:**

Addressing the issue of fruit recognition and localization failures in harvesting robots due to severe occlusion by branches and leaves in complex orchard environments, this paper proposes an occlusion avoidance method that combines a lightweight YOLOv8n model, developed by Ultralytics in the United States, with active perception. Firstly, to meet the stringent real-time requirements of the active perception system, a lightweight YOLOv8n model was developed. This model reduces computational redundancy by incorporating the C2f-FasterBlock module and enhances key feature representation by integrating the SE attention mechanism, significantly improving inference speed while maintaining high detection accuracy. Secondly, an end-to-end active perception model based on ResNet50 and multi-modal fusion was designed. This model can intelligently predict the optimal movement direction for the robotic arm based on the current observation image, actively avoiding occlusions to obtain a more complete field of view. The model was trained using a matrix dataset constructed through the robot’s dynamic exploration in real-world scenarios, achieving a direct mapping from visual perception to motion planning. Experimental results demonstrate that the proposed lightweight YOLOv8n model achieves a mAP of 0.885 in apple detection tasks, a frame rate of 83 FPS, a parameter count reduced to 1,983,068, and a model weight file size reduced to 4.3 MB, significantly outperforming the baseline model. In active perception experiments, the proposed method effectively guided the robotic arm to quickly find observation positions with minimal occlusion, substantially improving the success rate of target recognition and the overall operational efficiency of the system. The current research outcomes provide preliminary technical validation and a feasible exploratory pathway for developing agricultural harvesting robot systems suitable for real-world complex environments. It should be noted that the validation of this study was primarily conducted in controlled environments. Subsequent work still requires large-scale testing in diverse real-world orchard scenarios, as well as further system optimization and performance evaluation in more realistic application settings, which include natural lighting variations, complex weather conditions, and actual occlusion patterns.

## 1. Introduction

With the increasing shortage of agricultural labor and the aging population, the development of artificial intelligence and robotics has made the realization of automated and intelligent harvesting robots possible, rendering them an urgent demand for modern agriculture [1,2,3,4]. In orchard environments, efficient harvesting operations rely on robots equipped with advanced visual perception systems. These systems enable rapid and accurate identification of fruit position and posture in complex and variable scenarios [5,6], thereby facilitating optimal picking path planning. This approach enhances production efficiency and product quality while minimizing crop damag.

Visual perception serves as the “eyes” of harvesting robots, forming the core component for target recognition and precise positioning. However, in complex field environments, severe occlusion of fruits by branches and leaves can significantly compromise the reliability of visual systems. Occlusion results in incomplete target feature information, making it difficult for traditional passive vision methods to accurately identify and locate targets. This can even lead to picking failures, severely limiting the practical application and widespread adoption of harvesting robots [7].

To address the challenge of occlusion, researchers have proposed various solutions. In the domain of passive perception, deep learning methods have been widely adopted: Iñiguez et al. investigated the effects of occlusion by analyzing pixel correlations at different defoliation stages [8]; Gené-Mola et al. compared the accuracy of point cloud methods for apple size estimation under different occlusion conditions [9]; The ORCNN model proposed by Follmann et al. can predict “amodal masks” for occluded parts to estimate complete targets [10]; Sun et al. employed Soft-NMS to mitigate the decline in bounding box confidence caused by occlusion [11]. Although these methods enhance model robustness, their performance remains constrained by the information completeness of the initial viewpoint. 

In recent years, active sensing has emerged as a cutting-edge approach to addressing occlusion challenges due to its advantage of “trading motion for information” [12]. This technology treats the robot as a closed-loop intelligent agent integrating “perception-decision-action”: when the initial view provides insufficient information, the system can reason and actively plan optimal observation actions (such as moving the camera or robotic arm) to acquire new perspectives with higher information gain. This enables comprehensive understanding of targets and fundamentally avoids permanent occlusion inherent in fixed viewpoints [13]. 

Despite its advanced concept, existing active perception methods still face several challenges. First, they suffer from low exploration efficiency—many systems employ fixed trajectories (e.g., spiral or grid patterns) or multi-viewpoint traversal, resulting in excessive redundant movements and prolonged time consumption, which severely impacts operational efficiency [1]. Second, the decision-making intelligence is often insufficient, as simply following gradients or predefined paths tends to trap the system in local optima. Additionally, the real-time performance bottleneck of front-end perception modules is particularly prominent: active perception is a high-frequency iterative closed-loop process, where each cycle must be completed within an extremely short timeframe. If the visual algorithms used for occlusion evaluation and decision generation involve heavy computations and high latency, the response speed of the system will be severely constrained, making exploration efficiency inadequate for practical operational demands. Consequently, existing systems often compromise between “recognition accuracy” and “exploration time,” struggling to achieve both high success rates and high efficiency. 

In terms of implementation approaches, researchers have attempted to improve efficiency. For example, Zapotezny-Anderson and Lehnert designed a multi-camera matrix to capture multi-angle images of the same target, constructing an occlusion variation dataset [14]. They trained a CNN model to directly estimate the spatial gradient of the occlusion rate from a single initial image, indicating the direction in which the camera should move to most effectively reduce occlusion. This method achieves a direct mapping from “perception” to “action” and possesses certain real-time potential. However, its effectiveness still heavily relies on whether the front-end perception model can rapidly and accurately assess the state—a high-latency perception model would completely negate the advantages of this “direct mapping,” and even with an optimal decision-making strategy, the overall system efficiency would remain limited. 

In summary, existing research primarily focuses on improving recognition efficiency under occlusion conditions and relies on multi-view information acquisition. However, there remains a gap in meeting the stringent real-time requirements of active perception. An ideal solution must first break through the real-time bottleneck at the perception end to support efficient exploration. To this end, this study focuses on constructing a lightweight object detection model that satisfies the real-time demands of active perception. Based on YOLOv8n, this paper introduces the C2f-FasterBlock module and the SE (Squeeze-and-Excitation Networks) attention mechanism to develop a lightweight model characterized by high accuracy, low latency, and easy deployment. The aim is to provide robust real-time perceptual support for robots to rapidly and accurately assess occlusion states and make informed decisions. 

## 2. Materials and Methods

### 2.1. Active Perception Strategy and Model Construction

#### 2.1.1. Active Perception Process

The active perception method adopted in this study utilizes a fruit harvesting robot as the primary platform, with its core workflow as follows: First, from the current observation angle, the lightweight YOLOv8n model proposed in this paper is employed to obtain the target recognition confidence (C), while combining deep learning and image processing methods to calculate the occlusion rate (ρ_o_) (specific calculation method detailed in Section 2.1.2). When the confidence falls below a set threshold (0.6) or the occlusion rate exceeds a threshold (0.4), the system determines that the current viewpoint provides insufficient information and initiates the active perception cycle. 

During the active perception cycle, the system inputs the current observation image into a pre-trained deep learning network based on ResNet50 and multi-modal fusion. This network is trained to directly predict the optimal movement direction for the robotic arm’s end-effector. Upon obtaining the predicted direction, the robotic arm executes the corresponding movement. The directional command is transmitted as a 3D displacement vector via a ROS topic to the robotic arm’s low-level controller, driving fine adjustments in Cartesian space. Simultaneously, the front-end camera continuously captures images from new perspectives, which are processed in real-time by the lightweight YOLOv8n model for object detection. The detection confidence score is published to the control system via a ROS message. The control system monitors this confidence score in real-time and immediately sends a stop command to the motion module once a predefined target threshold (≥0.8) is reached, terminating the robotic arm’s movement. At this stage, the target fruit is considered sufficiently exposed and reliably localized, prompting the system to proceed to the next phase for precise picking operations.

#### 2.1.2. Construction of Dataset

Accurate calculation of the occlusion rate forms the foundation for active perception decision-making. This paper adopts a combined approach of deep learning and image processing methodologies. Initially, the instance segmentation capability of the lightweight YOLOv8n model is utilized to extract the region of interest (ROI) of the target fruit. Subsequently, two complementary methods are employed to calculate the occlusion rate: 

Method based on color thresholding and circular fitting: The ROI is converted to the HSV color space, and fruit pixels are extracted using empirically determined thresholds that capture the dominant red–yellow hues of ripe apples commonly found in commercial orchards:

$$\left\{\begin{array}{c} H\in [0,15]\cup [165,180]\\ S\in [50,255]\\ V\in [50,255] \end{array}\right.$$

These ranges were calibrated based on a representative set of 30 artificial apple models spanning typical maturity stages (from partially green to fully red) used in our simulated orchard environment. While this range effectively isolates mature red/yellow apples from green foliage under controlled indoor lighting, it may not generalize well to unripe green apples or apples under strong natural illumination variations (e.g., overcast vs. direct sunlight). This limitation is acknowledged in Section 4.4 and will be addressed in future field trials with real fruits across diverse ripeness levels.

(1) $$\rho_{o}=min\left(1,1-k\frac{\sum P_{f}}{\sum P_{circle}}+\alpha_{r1}\right)$$

In Equation (1), $P_{f}$ represents the fruit area, k denotes the correction coefficient, and $\alpha_{r1}$ represents the correction value used to compensate for the discrepancy between the calculated and actual values. Due to variations in fruit size, which are primarily approximate ellipses, $P_{circle}$ represents the minimum enclosing circle area calculated based on the fruit region. If the calculated value of $\rho_{o}$ is less than 1, it is set to 1.

**Figure 1:**
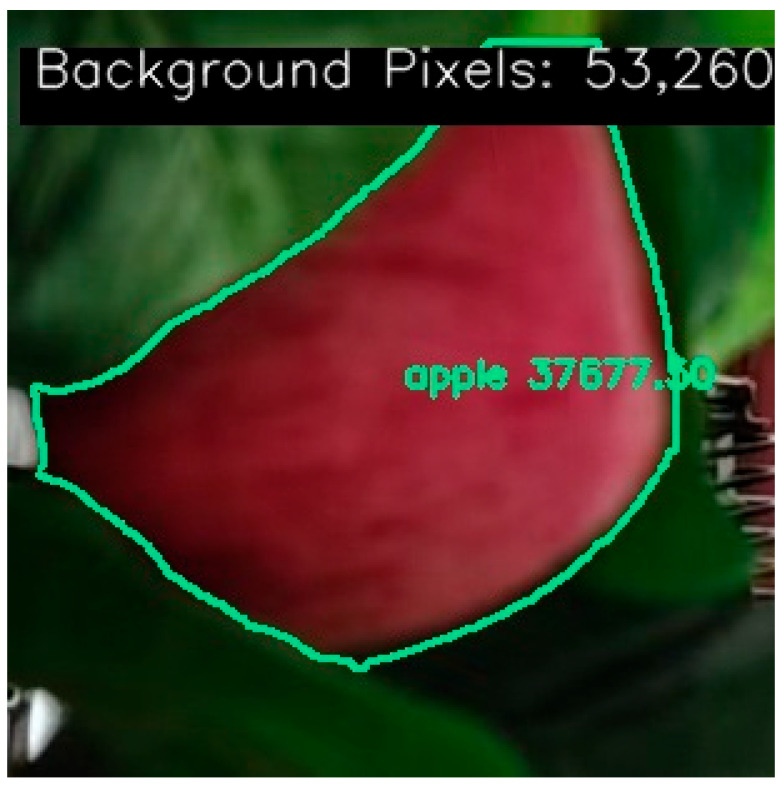
Pixel values of target and background regions obtained using instance segmentation function.

Method based on pixel-level statistics using semantic segmentation masks: Firstly, the binary mask generated by instance segmentation is utilized to count pixel values in the target area and background area (as shown in Figure 1). The occlusion rate is then derived by incorporating a deviation factor for correction.

(2) $$\rho_{o}=\frac{\sum \left(px=0\right)}{\sum \left(px=0\right)+\sum \left(px=1\right)}+\alpha_{r2}$$

(3) $$\alpha_{r2}=\alpha_{r2}*\left(0.6-\rho_{o}\right)$$

In Equation (2), $\sum \left(px=1\right)$ represents the pixel values in the apple region, $\sum \left(px=0\right)$ represents the total pixel values in the background region, and $\alpha_{r2}$ is the deviation factor with an initial value of 0.1. Its value decreases as the occlusion ratio increases and becomes negative when a certain threshold is exceeded. Additionally, occlusion rates below a specific threshold (e.g., 6%) are considered negligible, and values where $\rho_{o}$ < 6% are set to 0.1%. 

To reduce estimation errors from individual methods, this study employs weighted fusion of the results from both approaches (with weights of 0.6 and 0.4, respectively) to enhance overall accuracy. This weight distribution was determined through preliminary experimental analysis. A grid search was conducted on a small-scale validation set containing 30 samples with varying occlusion levels and lighting conditions. Different weight combinations ranging from 0.1:0.9 to 0.9:0.1 (in increments of 0.1) were evaluated using the Root Mean Square Error (RMSE) between the fused occlusion rate and the ground truth, which was established through meticulous manual annotation. Experimental results demonstrated that the 0.6:0.4 weight combination achieved the lowest RMSE, thus providing the optimal balance for the fusion strategy.

**Figure 2:**
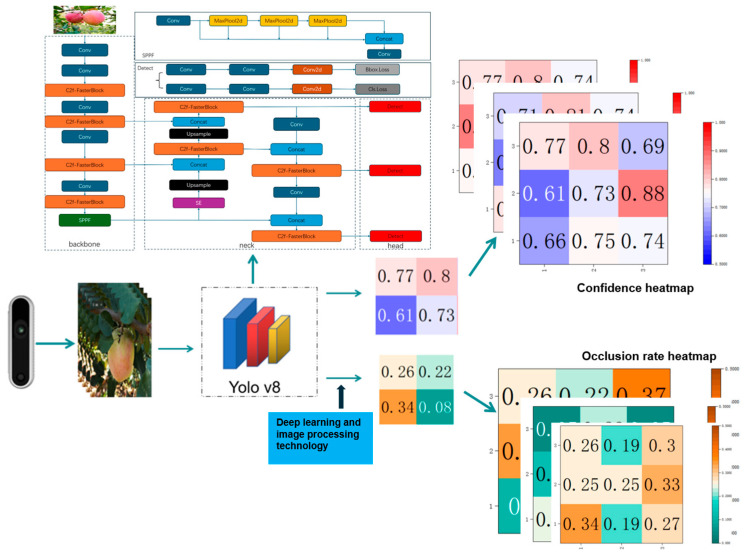
Production process of active perception dataset.

After obtaining the occlusion rate and confidence score at the initial position, the robot uses this location as a starting point to move the camera within a predefined three-dimensional spatial matrix, sampling multiple surrounding positions and recording the data from each location. Specifically, this 3D spatial matrix is a discrete grid of size 5 × 5 × 3 centered on the current target fruit, corresponding to a physical space range of 15 cm × 15 cm × 3 cm, with uniform sampling at 3 cm intervals along each axis. The sampling strategy adopts a systematic traversal approach, where the robotic arm, with the camera mounted on it, sequentially moves to each grid point to capture images and calculate the corresponding recognition confidence score and occlusion rate, thereby constructing confidence and occlusion rate distributions. Finally, by analyzing the distribution characteristics, the optimal waypoint is determined, from which the optimal movement direction is derived. This direction, together with the initial image, forms a training sample pair of “image, optimal movement direction”. The dynamic matrix dataset constructed in this way encapsulates the dynamic relationship between occlusion states and spatial positions. The main workflow is illustrated in Figure 2.

Through the aforementioned method, this paper constructed an active perception training dataset comprising 600 sample pairs. Each pair consists of an initial RGB observation image and its corresponding optimal robotic arm movement direction vector. Limited by the current experimental platform conditions, all data were collected in a simulated indoor orchard environment: artificial apple models (55–90 mm in diameter) were fixed on artificial branch-and-leaf structures, and a 6-DOF robotic arm equipped with a Realsense D455 camera performed multi-angle observations within a predefined 3D spatial grid to replicate occlusion variations from different viewpoints.

Although the data was acquired in a controlled indoor lighting environment and did not cover complex illumination conditions found in natural orchards (such as strong backlighting, overcast, or cloudy diffuse light), an image augmentation strategy was implemented during training to enhance the model’s robustness to visual disturbances in practical applications. Augmentations included: simulated lighting variations and noise injection. These enhancement techniques effectively expanded the distribution diversity of the input data without altering occlusion semantics, helping to mitigate overfitting risks caused by the homogeneity of the indoor collection environment and enhancing the model’s generalization capability across different lighting conditions. 

#### 2.1.3. Active Perception Model

This paper adopts an end-to-end visual trajectory prediction model based on ResNet50 and multi-modal fusion as the decision-making core within the active perception framework. The model is designed to directly regress the future movement direction of the robotic arm’s end-effector from a single RGB image. 

In the “perception-decision-action” closed loop of active perception, the real-time performance and deployability of the front-end perception module directly determine the system’s practicality in real agricultural scenarios. Each decision cycle relies on fast and accurate analysis of the current observed image. If the perception stage suffers from high latency or an overly large model, even the most intelligent decision-making network would be constrained by the system’s overall response speed, making it difficult to deploy on resource-constrained embedded devices or robot controllers. Therefore, a highly real-time, lightweight, and easily deployable front-end detection model is an indispensable prerequisite for building an efficient and practical active perception system. The lightweight YOLOv8n model constructed in the next section is specifically designed to meet this critical requirement. This model achieves an extremely low parameter count and a compact model volume, not only delivering an inference speed of up to 83 FPS for rapid state evaluation but, more importantly, effectively reducing the demand for computational resources and storage space. This makes it more suitable for practical deployment and application on edge computing platforms such as agricultural robots. 

**Figure 3:**
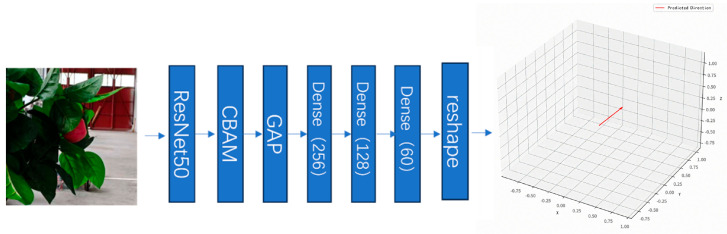
Active Perception Network Structure.

Based on this foundation, the decision-making model in this study adopts a two-stage architecture of “feature extraction-mapping regression.” The front-end employs a pre-trained ResNet50 network as the visual encoder to derive high-level semantic features from the input image. After resizing to standard dimensions and normalization, the input image is fed into the ResNet50 network. The feature map output from its final convolutional layer is compressed by a global average pooling (GAP) layer into a 2048-dimensional vector, serving as the global embedded representation of the image. To preserve pre-trained knowledge and accelerate convergence, the parameters of the ResNet50 network remain frozen during training. The back-end consists of a three-layer fully connected network that functions as the trajectory decoding head, responsible for mapping the visual embedding into movement direction information in three-dimensional space. This architecture achieves direct mapping from visual perception to motion planning. The model uses the mean squared error (MSE) as the loss function and is trained with the Adam optimizer. The structure of the active perception network is shown in Figure 3. 

This paper employs the Mean Squared Error (MSE) as the model’s loss function. The core task of this active perception model is a typical regression problem: based on the current observation image, it predicts the movement direction information of the robotic arm’s end-effector over future consecutive time steps. The MSE loss effectively quantifies the discrepancy between the predicted direction and the true direction, and drives model parameter optimization via gradient descent algorithms.

For a given training sample, let the true trajectory be $y=[y_{1},y_{2},\ldots ,y_{9}]\in R^{9}$, where this vector contains the (x, y, z) three-dimensional coordinates for the next three time steps in chronological order. Correspondingly, the model’s predicted direction vector is $\hat{y}=[{\hat{y}}_{1},{\hat{y}}_{2},\ldots ,{\hat{y}}_{9}]\in R^{9}$. The loss function for this single sample is defined as the mean of the squared differences between the predicted and true values across all dimensions, calculated as follows:

(4) $$L_{MSE}=\frac{1}{9}\sum_{i=1}^{9}\;{\left(y_{i}-{\hat{y}}_{i}\right)}^{2}$$

In practical training, models are typically optimized in batches. For a training batch of size ***B***, the overall loss L is computed as the arithmetic mean of the losses for all samples within the batch:

(5) $$L=\frac{1}{B}\sum_{b=1}^{B}\;L_{MSE}^{\left(b\right)}$$

During the training process, the loss function L serves as a quantitative metric of model performance. After the loss value is computed via forward propagation, the model uses the backpropagation algorithm to calculate the gradients of the loss function with respect to the parameters of each network layer. Subsequently, the Adam optimizer is employed to update the network weights based on these gradients. This iterative process aims to minimize the loss function L progressively, thereby causing the model’s predicted movement direction $\;\hat{y}$ to continuously approach the true direction y, effectively fitting the optimal mapping function from visual perception to motion planning. 

### 2.2. Construction of Lightweight Models

#### 2.2.1. Lightweight YOLOv8n Model

This study employs an active perception approach to mitigate occlusion effects during target recognition for harvesting robots. However, both the model construction phase and operational phase require YOLO-based detection: initially for target recognition and segmentation to generate training datasets, and subsequently for real-time detection to determine the robotic arm’s termination position. Consequently, a lightweight model with high detection speed and easy deployment is essential to facilitate both dataset construction for active perception training and real-time localization during implementation. 

**Figure 4:**
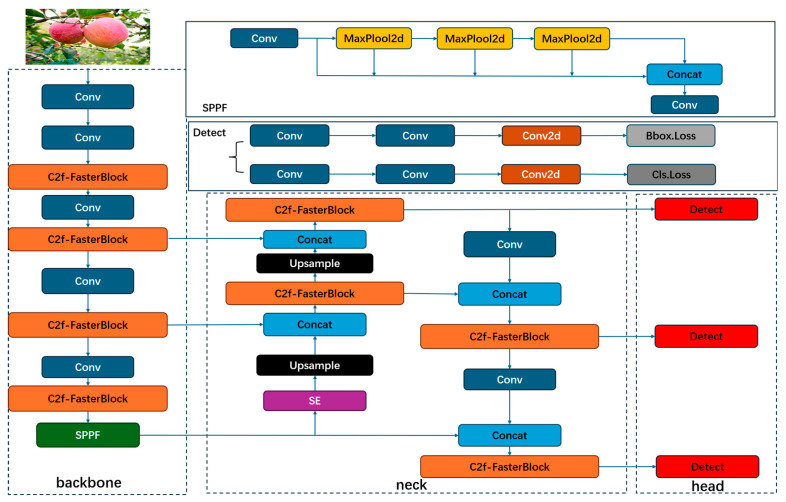
The structure diagram of the improved YOLOv8 model in this paper. Modules with different functionalities are distinguished by different colors.

To meet the aforementioned requirements, this paper proposes a lightweight YOLOv8n model based on the FasterBlock module and SE (Squeeze-and-Excitation Networks) attention mechanism, with its overall architecture shown in Figure 4. The model follows the standard structure of YOLOv8, consisting of three components: the Backbone, the Neck, and the Head. 

In the backbone and neck networks, this study replaces the Bottleneck structure in the original C2f module with the FasterBlock module from FasterNet. This modification effectively reduces the model’s parameter count and memory access overhead, enhancing inference speed while decreasing the model size, thereby making it more suitable for deployment on resource-constrained embedded platforms. Furthermore, through its unique design, the FasterBlock module helps the model focus on the salient central regions of the target, thereby better preserving critical feature information. 

To further compensate for potential accuracy loss due to model lightweighting and enhance the model’s ability to extract important features, this study introduces the SE attention mechanism into key layers of the network. This mechanism adaptively learns the importance weights of each feature channel, strengthening the expression of critical features while suppressing irrelevant or redundant background interference, with almost no additional computational overhead. Consequently, it maintains high efficiency while preserving or even improving the model’s detection accuracy [15,16]. 

Although YOLOv11, as the latest official version from Ultralytics, demonstrates superior detection performance, this paper still selects YOLOv8n as the base model, primarily based on the following practical considerations. First, the core objective of this research is not to pursue state-of-the-art detection accuracy, but to construct a lightweight perception module characterized by high real-time performance, low resource usage, and ease of deployment on embedded agricultural robot platforms, thereby supporting high-frequency closed-loop active perception. YOLOv8n already offers a good balance between model size, inference speed, and accuracy. Furthermore, its architecture is concise with robust community support, facilitating modular improvements (such as integrating the C2f-FasterBlock and SE attention mechanisms). Second, although YOLOv11 exhibits better performance on standard datasets, its default model complexity is higher. Without targeted lightweight optimization, it would be difficult to meet this system’s stringent requirements for latency and power consumption. Additionally, to ensure experimental reproducibility and fair comparison, all ablation studies and comparative experiments with other lightweight models like YOLOv9t in this paper were conducted within a unified technical stack (the Ultralytics YOLOv8 framework). In future work, we will explore migrating the lightweight strategies proposed herein to newer frameworks like YOLOv11 to further enhance system performance.

#### 2.2.2. C2f-FasterBlock

**Figure 5:**
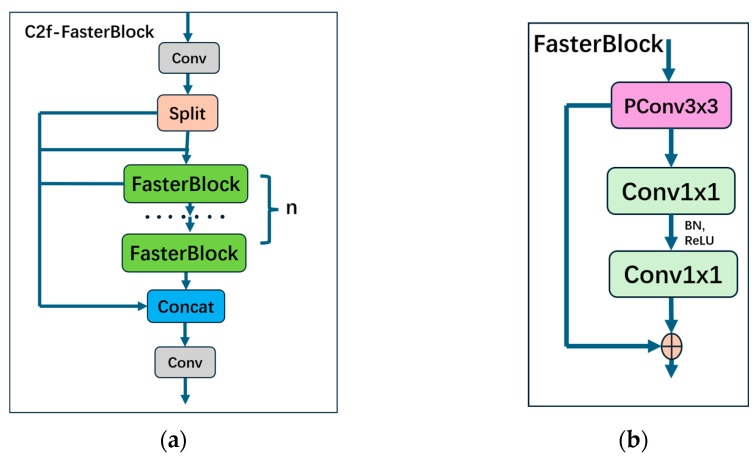
(**a**) C2f-FasterBlock module structure; (**b**) FasterBlock modulestructure. Modules with different functionalities are distinguished by different colors.

This paper proposes a lightweight feature learning module named C2f-FasterBlock (the structure is shown in Figure 5a), designed based on the FasterBlock module, to replace the original C2f module in YOLOv8n. The FasterBlock module is an efficient and lightweight neural network component, whose core concept is to optimize computational methods, thereby enhancing inference speed while maintaining robust feature extraction capabilities. 

As shown in Figure 5b, the core of the FasterBlock module is Partial Convolution (PConv) [17]. This operation divides the channels of the input feature map into two groups: one group undergoes standard depthwise convolution to extract spatial features, while the other group retains the original values. Subsequently, these two sets of features are fused through concatenation. This design reduces the model’s computational load (GFLOPs) and memory access overhead while avoiding the loss of critical spatial information. 

Following PConv, the module employs Pointwise Convolution (PWConv) to enable cross-channel information interaction and incorporates Residual Connections to facilitate gradient flow in deep networks. The overall design is concise, efficient, and hardware-friendly, making it particularly suitable for mobile or real-time application scenarios. 

**Figure 6:**
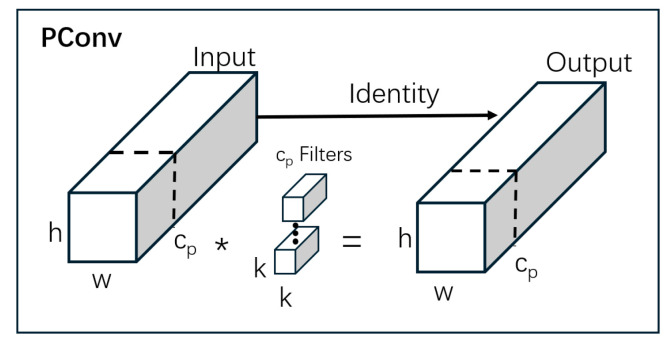
Pconv. The asterisk symbol “*” represents the channel-wise multiplication operation.

By integrating the FasterBlock module, the C2f-FasterBlock module significantly reduces computational complexity and memory consumption while effectively improving real-time inference performance. More importantly, its unique structural design ensures the model’s original feature extraction capability is fully preserved, providing strong support for achieving model lightweighting and high-speed performance without sacrificing detection accuracy. The detailed structure of Partial Convolution (PConv) is illustrated in Figure 6. 

#### 2.2.3. Squeeze-and-Excitation Networks

**Figure 7:**
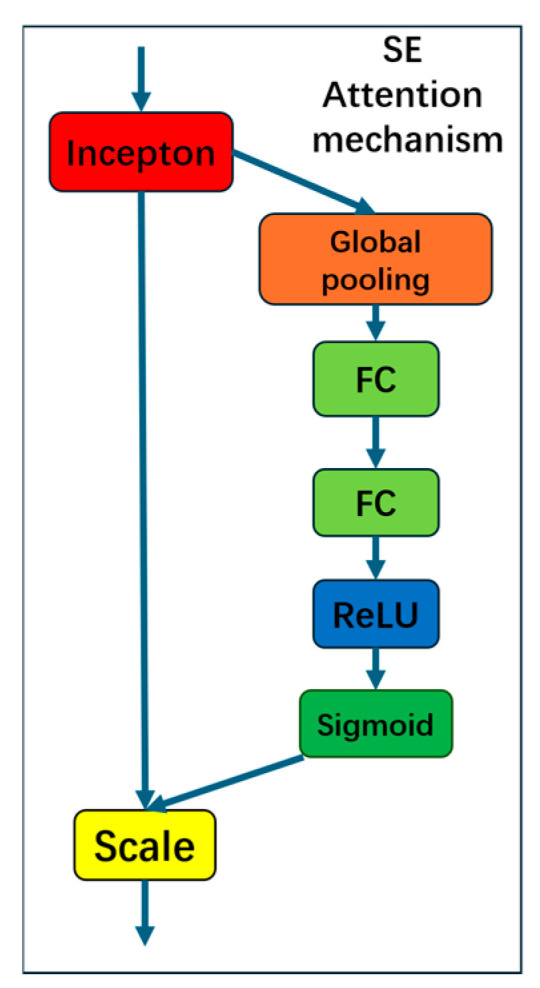
Structure of the SE attention mechanism. Modules with different functionalities are distinguished by different colors.

The SE (Squeeze-and-Excitation) attention mechanism adopted in this study is a highly lightweight channel attention module, whose core advantage lies in significantly improving network performance with minimal computational cost. This mechanism explicitly models interdependencies between channels in convolutional feature maps through a three-step “Squeeze-Excitation-Scale” operation: First, global average pooling compresses spatial information from each channel into a scalar descriptor (Squeeze). Next, a compact MLP network learns importance weights for each channel (Excitation). Finally, the obtained weights are multiplied back to the original feature map channel-wise, enhancing responses from important channels while suppressing redundant ones (Scale). After incorporating the SE attention mechanism, the model demonstrates significantly improved focus on critical feature channels, while the introduced additional parameters and computational cost (GFLOPs) are nearly negligible. Consequently, this mechanism not only enhances detection accuracy but also effectively meets the lightweight requirements of our model. The specific structure of the SE attention mechanism is illustrated in Figure 7. 

## 3. Experimental Design and Evaluation Indicators

### 3.1. Experimental Design

All network training and testing were conducted on a PC equipped with an Intel^®^ Xeon^®^ Silver 4210R CPU and an NVIDIA GeForce RTX 3090 GPU. The models were built using PyTorch 1.12.1 within a virtual environment created by Anaconda3, configured with Python 3.10 and CUDA 11.3. The training parameters are as follows: 

Training parameters for the lightweight model: input image size 640 × 640, training epochs 300, batch size 16. 

Training parameters for the active perception model: optimizer Adam, initial learning rate 1 × 10^−4^, batch size 16, total training epochs 15. The loss function uses Mean Squared Error (MSE Loss) to measure the difference between the predicted movement direction and the true optimal direction. 

The training dataset for the active perception model is a dynamically constructed matrix dataset generated during the robot’s active exploration in an apple orchard (as described in Section 2.1.2). This dataset comprises numerous “initial observation image, optimal movement direction” samples, where the initial observation image is an RGB image and the optimal movement direction is a displacement vector in 3D space. To ensure training reliability and unbiased evaluation, the dataset was partitioned into training and validation sets using stratified random sampling with an 8:2 ratio. The 80% training set and 20% validation set split is a widely adopted strategy in machine learning that balances efficiency and reliability. It ensures the model has sufficient samples for effective learning while retaining an adequately sized, independent validation set for stable generalization performance assessment. This ratio is well-suited for most medium-sized datasets, is straightforward to implement, incurs low computational overhead, and facilitates rapid iteration and hyperparameter tuning. The training set is used for learning and optimizing model parameters, while the validation set is used to periodically evaluate performance on unseen data during training, monitor for overfitting, adjust hyperparameters, and select the optimal model.

After model training was completed, tests were conducted on target apples under varying degrees of occlusion in a simulated orchard environment set up in the laboratory. The experimental platform consisted of a six-degree-of-freedom robotic arm equipped with a RealSense D455 depth camera. The experimental procedure strictly adhered to the active perception framework: 

State assessment: The robotic arm moves to the initial observation position to capture an image. 

Decision triggering: The lightweight YOLOv8n model is utilized for detection to evaluate target confidence C and occlusion rate ρ_o_. 

Active exploration: If C < 0.6 or ρ_o_ > 0.4, initiate the loop: input the current image into the trained active perception network to predict the optimal movement direction, execute the robotic arm’s movement, and output real-time detection results to control the termination of the robotic arm’s motion. 

### 3.2. Evaluation Metrics for Network Models

This study selects mean Average Precision (mAP), Frames Per Second (FPS), and computational complexity (GFLOPs) as the primary evaluation metrics. AP is a composite metric that measures model performance by calculating the area under the Precision (P) and Recall (R) curve. Specifically, the accuracy of model predictions is determined by the Intersection over Union (IoU) between ground truth and predicted values. The relevant formulas are as follows:

(6) $$P=\frac{TP}{TP+FP}$$

(7) $$R=\frac{TP}{TP+FN}$$

(8) $$AP=\sum_{i=1}^{N}\;P\left(i\right)\Delta R\left(i\right)$$

It is worth noting that for each IoU threshold, *AP* is calculated based on the cumulative *TP*, *FP*, and *FN* of all test samples. This study uses mAP as the primary evaluation metric. mAP is the average of *AP* values across a series of different confidence thresholds (typically ranging from 0.5 to 0.95 with an interval of 0.05). FPS (Frames Per Second) refers to the number of image frames processed per second, which is a critical metric for evaluating the real-time performance of the model. A higher FPS value indicates faster processing speed and better real-time performance. GFLOPs (Giga Floating Point Operations Per Second) is a key metric for assessing the computational complexity of the model, used to evaluate the total computational load required for a single forward inference. Lower GFLOPs indicate higher real-time monitoring speed and a higher degree of model lightweighting. 

## 4. Results

### 4.1. Active Perception Experiment

After completing model training, we conducted comprehensive performance tests on the proposed lightweight YOLOv8n model and the ResNet50-based active perception network. The aim was to validate the system’s autonomous decision-making capability, exploration efficiency, and final recognition success rate under complex occlusion conditions. 

**Figure 8:**
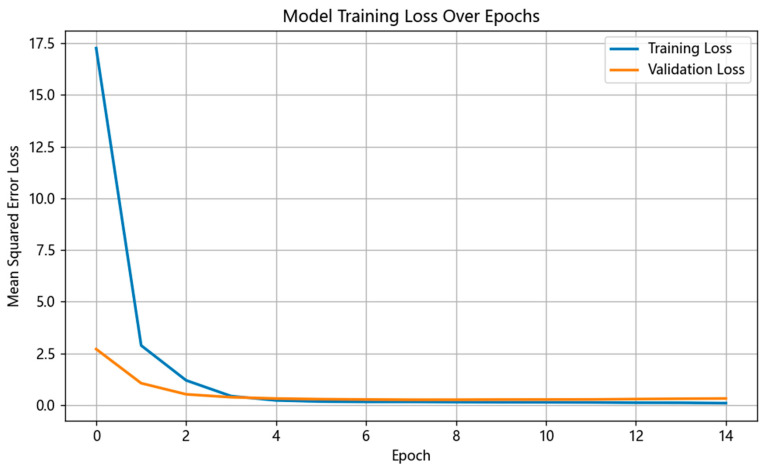
Variation in loss function (MSE Loss) on the training and validation sets of the active perception model.

Figure 8 shows the loss function (MSE Loss) curves of the active perception model on the training and validation sets. As illustrated, as the number of training epochs increases, both the training loss and validation loss decrease steadily and show no significant divergence after convergence, indicating a stable learning process, successful convergence, and no obvious overfitting. This establishes a solid foundation for subsequent testing. 

**Figure 9:**
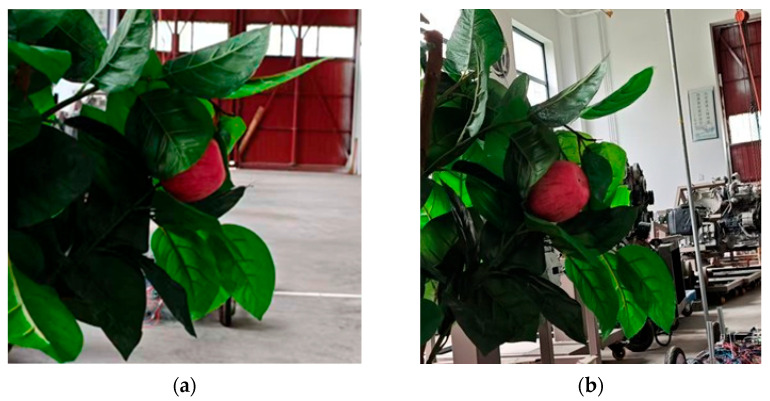
Simulation experiment results (**a**) Initial observation position image; (**b**) De Optimal observation image obtained after active perception.

Figure 9 presents the experimental results of the active perception model. Figure 9a shows the initial observation where the target fruit is severely occluded, resulting in low detection confidence by the lightweight YOLOv8n model, which fails to meet reliable recognition requirements. Figure 9b displays the optimal observation obtained after one active exploration cycle, demonstrating effective avoidance of occlusions with clear and complete fruit contours. The detection confidence is significantly improved while the occlusion rate is reduced, fully meeting the positioning accuracy requirements for harvesting operations. 

### 4.2. Comparative Experiments of Different Models

To systematically evaluate the performance of the proposed lightweight YOLOv8n network in image detection tasks, this study compares the model with the original YOLOv8 models of different scales (e.g., n/s/m). Evaluation metrics include mAP, FPS, GFLOPs, the number of model parameters, and model weight file size, comprehensively assessing the model’s detection accuracy and real-time performance. Table 1 summarizes the performance comparison results of the models. 

As shown in Table 1, compared to the original YOLOv8n algorithm, the proposed method reduces the number of parameters and computational complexity while improving the model’s detection accuracy (mAP increased by 3%). Furthermore, the proposed model demonstrates superior detection performance relative to other, more complex variants of YOLOv8. Additionally, when compared to the existing lightweight model YOLOv9t, the proposed algorithm exhibits a slight increase in parameter count but outperforms the former in terms of detection accuracy, model weight file size, and computational complexity. Therefore, based on the comparative experimental results, it can be concluded that the lightweight design of the proposed algorithm is successful.

### 4.3. Ablation Study

Through ablation experiments on the apple detection dataset, this study systematically evaluated the effectiveness of each proposed improvement module in the lightweight YOLOv8n network. Using the original YOLOv8n network as the baseline, the experiments progressively incorporated two innovative modules: C2f-FasterBlock and the SE attention mechanism. The ablation experiments examined the impact of each module on the accuracy of apple target recognition and localization, as well as their effectiveness in model lightweighting and inference acceleration. Table 2 summarizes the performance of the models with different improvement modules incorporated. 

As shown in Table 2, after incorporating the C2f-FasterBlock module, the network’s parameter count and computational load show a significant reduction (computational load decreased by 42%, parameter count decreased by 37%). This is attributed to the module’s characteristic of performing depthwise convolution only on partial channels of the input feature map. After integrating the SE attention mechanism, the mAP for apple detection increased by 2% compared to the baseline model, demonstrating its enhanced focus on key feature channels and improved capability to handle targets of varying sizes. The experimental results indicate that the proposed modules effectively improve detection accuracy while significantly enhancing real-time detection speed and reducing computational complexity.

### 4.4. Research Limitations 

Although this study has made certain progress in active perception strategies and lightweight object detection models, several key limitations remain and need to be addressed in future research:

The current experiments were primarily conducted in a laboratory setting, where the test scenarios were relatively simple and occlusion patterns were relatively uniform. Although various occlusion scenarios were constructed using simulated occluders, real orchard environments present far more complex and variable occlusion conditions, including intertwined branches/leaves and varying lighting. This idealized experimental environment may not fully reflect the method’s true performance in practical applications, limiting the generalizability of the findings.

Due to the reliance of the current experimental platform on fixed power supply, the harvesting robot lacks an independent mobile power system, preventing mobile, long-duration field tests in real orchard environments. Consequently, this study could not systematically evaluate the method’s performance across different times of day (morning/noon/dusk), different weather conditions (sunny/overcast/rainy/foggy), or different fruit maturity stages. The absence of these environmental factors prevents a comprehensive validation of the method’s robustness and adaptability in real-world applications, impacting the practical value of the conclusions.

It should be noted that the HSV-based occlusion estimation relies on fixed color thresholds optimized for the artificial apple models used in our lab setting, which primarily mimic mid-to-late maturity stages. The method does not explicitly adapt to color shifts caused by varying ripeness (e.g., green vs. red apples) or dynamic lighting conditions. In real orchards, such variability could lead to under-segmentation of unripe fruits or false positives from reddish leaves. Future work will incorporate adaptive color modeling or deep learning–based segmentation (e.g., Mask R-CNN) trained on multi-ripeness datasets to improve robustness.

The proposed active perception strategy was primarily validated against specific types of occlusion patterns. While it demonstrated good effectiveness, its generalizability across different occlusion types, target objects, and scenarios requires further verification. In particular, its effectiveness in complex occlusion scenarios (e.g., multi-object occlusion, dynamic occlusion) needs more thorough testing. 

As many advanced models in the field of active perception are either not open-source or lack detailed public documentation, this study could not conduct fair, reproducible quantitative comparisons with a broader range of state-of-the-art methods. Furthermore, the improvements made to the YOLO model in this work primarily targeted lightweight and real-time requirements, rather than specifically optimizing for occlusion handling. Therefore, the dimensions and objectives for comparison with detection models specifically designed for occlusion are not entirely aligned. 

The dataset scale remains relatively limited. Although public datasets and a self-constructed dataset were used, the overall data volume is relatively small, particularly regarding samples covering different environmental conditions and occlusion patterns. This somewhat constrains the model’s generalization ability and robustness. 

The aforementioned limitations primarily affect the practical application value and generalizability of this study’s conclusions. The lack of validation in real, complex environments prevents an accurate assessment of the method’s performance in actual orchard scenarios. The absence of testing under diverse environmental conditions restricts the evaluation of the method’s adaptability to different lighting and weather. Limitations in the scope of comparison mean the claimed advantages require validation against a wider range of methods. These limitations need to be addressed in future work through large-scale field tests and more comprehensive comparative experiments. 

## 5. Discussion and Future Work

### 5.1. Discussion

Aiming at the problem of fruit recognition failure in harvesting robots caused by severe occlusion in complex orchard environments, and addressing the urgent need to enhance the operational reliability and efficiency of agricultural robots through active perception technology, this paper proposes an occlusion avoidance solution based on the collaboration of a lightweight YOLOv8n model and an active perception network. Within the active perception framework, the real-time performance of the front-end perception module is crucial for efficient system operation. To this end, this paper constructs a lightweight and efficient YOLOv8n model. By introducing the C2f-FasterBlock module, the computational complexity (GFLOPs reduced to 5.1) and parameter count (37% reduction) are significantly decreased. Combined with the SE attention mechanism, the model effectively enhances the expressive capability of key features with almost no additional computational burden. Experimental results show that the model achieves an mAP of 0.885 in apple detection tasks, an FPS of 83, and a model size of only 4.3 MB, fully meeting the stringent requirements of the active perception system for real-time performance and lightweight design, thereby laying a solid foundation for rapid state assessment. 

It is worth delving deeper into the fact that the improvement in model performance fundamentally stems from a deliberate trade-off between accuracy and efficiency. The C2f-FasterBlock module reduces redundant computations by simplifying the network architecture, which is a key design choice for achieving real-time performance. However, this also means the model sacrifices some capability to capture subtle features. The introduction of the SE attention mechanism serves as a compensatory strategy for this trade-off—it enables the lightweight network to focus more on the key discriminative features of the fruit through dynamic weight allocation, thereby maintaining high detection accuracy despite a significant reduction in parameters. This design paradigm of a “lightweight backbone + strong focus” provides an effective solution for resource-constrained edge computing scenarios in agricultural robotics. 

However, the proposed solution still has clear boundaries of applicability. First, the model inherently relies on color and texture contrast. Performance may degrade when the fruit and background colors are highly similar (e.g., unripe green fruits against dense foliage) or under extreme lighting conditions. Second, the current experiments were primarily validated in controlled environments. The robustness of the closed-loop control of the active perception module in the face of dynamic disturbances in unstructured natural environments (such as swaying branches or sudden changes in lighting) still requires further validation through large-scale field trials. Lastly, the transition from static detection to dynamic interaction essentially involves sacrificing the utmost precision of single-frame analysis in exchange for enhanced overall system performance through multi-view fusion and decision optimization. This system-level trade-off necessitates targeted optimization based on specific operational scenarios during practical deployment. 

In summary, the proposed collaborative scheme of lightweight detection and active perception achieves efficient and reliable occlusion avoidance under an active perception paradigm. This approach not only excels in static detection but also demonstrates effective closed-loop control capabilities in dynamic interactions. The research provides a viable technical pathway for agricultural harvesting robots to achieve automated operations with high success rates and efficiency, showing promising application prospects.

### 5.2. Future Work

While this study has achieved interim results, some directions remain for deeper exploration due to experimental constraints and research progress. First, the current validation was primarily conducted in a simulated laboratory environment, lacking large-scale, multi-environment testing in real orchard conditions. The research team is dedicated to resolving challenges related to autonomous navigation and independent power supply for the harvesting robot’s mobile platform. Once the new generation of the mobile platform is developed, large-scale field experiments will be conducted immediately. These experiments will systematically evaluate the method’s performance under different times of day, weather conditions, and fruit maturity stages, comprehensively validating the method’s practical feasibility and robustness.

Second, it is important to note that this study primarily focuses on the innovative integration of a lightweight detection method with an active perception closed-loop framework, as well as the resulting system performance. However, the quantitative validation of the occlusion rate estimation method has not been fully explored. Specifically, the current work lacks a systematic validation of the occlusion rate estimation algorithm through manual annotation. Therefore, conducting detailed manual annotation will be the primary task in future research. By establishing a pixel-level occlusion annotation dataset, the accuracy of occlusion rate estimation can be quantitatively assessed, providing a more solid foundation for method optimization. This effort will further enhance the comprehensiveness of the validation framework for this solution. 

Furthermore, future work will investigate more complex occlusion scenarios, including multi-object occlusion, dynamic occlusion, and partial occlusion, to enhance the generalization capability of the active perception strategy. Simultaneously, we will explore the applicability of this method to various agricultural fruit harvesting scenarios, such as citrus and tomatoes, to verify its cross-crop generalizability. As research in related fields progresses, we will actively acquire more open-source models and datasets to conduct more comprehensive and fair comparative experiments, further validating the superiority of this method. Additionally, we will explore integrating this method with more advanced object detection models and active perception strategies to enhance overall performance. 

At the same time, efforts will be made to improve the system’s robustness to environmental factors such as lighting variations and weather changes, ensuring stable operation in actual orchard environments. By undertaking the tasks outlined above, we will further refine the theoretical framework and practical validation of this research, advancing the practical application of active perception technology in the field of agricultural robotics. 

### 5.3. Conclusions

This study proposes an occlusion avoidance framework integrating lightweight detection and active perception to address the challenge of fruit recognition caused by branch and leaf occlusions in complex orchard environments. The constructed lightweight YOLOv8n model achieves real-time detection at 83 FPS with a compact size of 4.3 MB, while maintaining high accuracy (mAP 0.885), meeting the requirements for embedded deployment. Combined with an end-to-end active perception network, the system enables dynamic viewpoint adjustment and closed-loop operation control, effectively improving recognition success rates and overall operational efficiency in occluded scenarios.

It should be noted that the current research is primarily validated in laboratory environments, and large-scale field trials in real orchard scenarios have yet to be conducted. The adaptability, stability, and generalization capability of the system under actual natural lighting, variable weather conditions, and complex unstructured environments require further validation and optimization through long-term, multi-scenario field testing.

This study provides a key technological pathway for the evolution of agricultural harvesting robots from static perception to dynamic interaction, embodying the intelligent system design philosophy of “compensating for visual limitations through active motion.” It lays a theoretical and methodological foundation for achieving high-success, high-efficiency autonomous operations in unstructured environments in the future. As the demand for agricultural automation continues to grow, such robust perception-decision integration technologies tailored to real-world complex scenarios will provide crucial support for the sustainable development of smart agriculture.

## Figures and Tables

**Table 1:** Comparison of experiments with different algorithms.

Network	mAP	Model Weight Size (MB)	GFLOPs	Parameters	FPS
YOLOv8n	0.854	6.3	8.9	3,157,200	66
YOLOv8s	0.875	22.6	28.5	11,166,560	69
YOLOv8m	0.877	52.1	79.3	25,902,640	40
YOLOv9t	0.828	4.7	7.6	1,974,684	70
Our network	0.885	4.3	5.1	1,983,068	83

**Table 2:** Comparison results of different object detection networks.

Network	C2f-FasterBlock	SE Attention Mechanism	mAP	Model Weight Size (MB)	GFLOPs	Parameters	FPS
YOLOv8n	×	×	0.854	6.3	8.9	3,157,200	69
	√	×	0.872	4.3	5.1	1,991,260	79
	×	√	0.875	6.3	8.1	3,017,740	72
	√	√	0.885	4.3	5.1	1,983,068	83

## Data Availability

Dataset available on request from the authors. The raw data supporting the conclusions of this article will be made available by the authors on request.

## References

[1] Sun T., Zhang W., Gao X., Zhang W., Li N., Miao Z. (2024). Efficient Occlusion Avoidance Based on Active Deep Sensing for Harvesting Robots. Comput. Electron. Agric..

[2] Zhang L., Jia J., Gui G., Hao X., Gao W., Wang M. (2018). Deep Learning Based Improved Classification System for Designing Tomato Harvesting Robot. IEEE Access.

[3] Jha K., Doshi A., Patel P., Shah M. (2019). A comprehensive review on automation in agriculture using artificial intelligence. Artif. Intell. Agric..

[4] Saleem M.H., Potgieter J., Arif K.M. (2021). Automation in agriculture by machine and deep learning techniques: A review of recent developments. Precis. Agric..

[5] Zhou H., Wang X., Au W., Kang H., Chen C. (2022). Intelligent robots for fruit harvesting: Recent developments and future challenges. Precis. Agric..

[6] Bai Q., Li S., Yang J., Song Q., Li Z., Zhang X. (2020). Object detection recognition and robot grasping based on machine learning: A survey. IEEE Access.

[7] Ji W., Zhang T., Xu B., He G. (2024). Apple recognition and picking sequence planning for harvesting robot in a complex environment. J. Agric. Eng..

[8] Iñiguez R., Palacios F., Barrio I., Hernández I., Gutiérrez S., Tardaguila J. (2021). Impact of Leaf Occlusions on Yield Assessment by Computer Vision in Commercial Vineyards. Agronomy.

[9] Gené-Mola J., Sanz-Cortiella R., Rosell-Polo J.R., Escolà A., Gregorio E. (2021). In-Field Apple Size Estimation Using Photogrammetry-Derived 3D Point Clouds: Comparison of 4 Different Methods Considering Fruit Occlusions. Comput. Electron. Agric..

[10] Follmann P., König R., Härtinger P., Klostermann M., Böttger T. Learning to See the Invisible: End-to-End Trainable Amodal Instance Segmentation. Proceedings of the IEEE Winter Conference on Applications of Computer Vision (WACV).

[11] Sun J., He X., Wu M., Wu X., Lu B. (2020). Detection of Tomato Organs Based on Convolutional Neural Network Under the Overlap and Occlusion Backgrounds. Mach. Vis. Appl..

[12] Ranganathan G.N., Apostolides P.F., Harnett M.T., Xu N.-L., Druckmann S., Magee J.C. (2018). Active Dendritic Integration and Mixed Neocortical Network Representations during an Adaptive Sensing Behavior. Nat. Neurosci..

[13] Li T., Wang C., Meng M.Q.-H., de Silva C.W. (2022). Attention-Driven Active Sensing with Hybrid Neural Network for Environmental Field Mapping. IEEE Trans. Autom. Sci. Eng..

[14] Zapotezny-Anderson P., Lehnert C. Towards Active Robotic Vision in Agriculture: A Deep Learning Approach to Visual Servoing in Occluded and Unstructured Protected Cropping Environments. Proceedings of the 1st IFAC Conference on Sensing, Control and Automation Technologies for Agriculture.

[15] Chen J., Kao S.-H., He H., Zhuo W., Wen S., Lee C.-H., Chan S.-H.G. Run, Don’t Walk: Chasing Higher FLOPS for Faster Neural Networks. Proceedings of the IEEE/CVF Conference on Computer Vision and Pattern Recognition (CVPR).

[16] Hu J., Shen L., Sun G., Albanie S. (2020). Squeeze-and-Excitation Networks. IEEE Trans. Pattern Anal. Mach. Intell..

[17] Molchanov P., Tyree S., Karras T., Aila T., Kautz J. (2016). Pruning Convolutional Neural Networks for Resource Efficient Transfer Learning. arXiv.

